# A Contextually Grounded Competence Framework for a Dental Education: A Multi-Method, Stakeholder-Informed Development Study

**DOI:** 10.3390/dj14060323

**Published:** 2026-05-29

**Authors:** Christina Gummesson, Liselotte Paulsson, Sofia Petrén, Nina Lundegren

**Affiliations:** 1 Faculty of Odontology, Malmö University, SE-205 06 Malmö, Sweden; liselotte.paulsson@mau.se (L.P.); sofia.petren@mau.se (S.P.); nina.lundegren@mau.se (N.L.); 2 Faculty of Medicine, Lund University, SE-221 00 Lund, Sweden

**Keywords:** competence framework, dental education, participatory design, decision-making, evidence-informed, collaborator, professional conduct

## Abstract

**Background/Objectives**: The dental profession is undergoing significant transformation driven by societal changes, technological advancements, evolving patient expectations, and increased attention to sustainability. These developments challenge traditional notions of dental competence and highlight the need for educational frameworks that support adaptability and longitudinal professional development. The aim of this study was to develop a contextually grounded competence framework for undergraduate dental education through an iterative, multi-method process informed by key educational stakeholders. **Methods**: A multi-method approach was used, combining a preparatory phase (literature review, interviews) with a development phase (drafting and workshops) that was revisited in response to feedback, followed by iterative voting rounds that prompted further minor revisions. A deductive exploratory mapping analysis aligned the emerging framework with existing intended learning outcomes across the curriculum. **Results**: The multi-method process produced descriptions of a framework that deliberately integrates roles, skills, and attributes to capture key dimensions of professional competence in dentistry. The framework includes six domains: ‘Evidence-informed’, ‘Decision-maker’, ‘Communicator’, ‘Acting with professional conduct’, ‘Health ambassador’, and ‘Collaborator and leader’. Across voting rounds, the domains were generally rated between ‘neutral’ and ‘very important’, with each round prompting minor revisions. Mapping suggested alignment between the overarching framework and the detailed curriculum. **Conclusions:** This study presents the outcome of a structured, exploratory multi-method process to develop a locally relevant competence framework, integrated into a dental education. The participatory design supported clarity and relevance. While sharing similarities with existing frameworks, the new framework also includes differences. The term ‘professional conduct’ was preferred rather than ‘professionalism’, and the domains ‘collaborator and leader’ and ‘decision-maker’ were identified as relevant according to employer expectations. Although the work was based locally at one dental school, the approach may be transferable to similar contexts.

## 1. Introduction

The dental profession is changing due to societal demands, organizational development in oral care, new technologies, and increased awareness of sustainability concerns. In society, there are shifts in sociodemography, with a growing proportion of older people, and expectations and demands on dental care are also evolving in relation to appearance and oral health. Consequences for dentists may include emerging ethical considerations and challenges, as well as an increased need to stay up to date with the best available evidence. Together, these developments urge a need for educational frameworks that can support adaptability to emerging challenges and local needs.

In Sweden, these societal developments and changing expectations have prompted us to re-examine which competencies are defined, developed, and assessed. One argument for developing new competency-based frameworks is the risk of a mismatch between previous curriculum emphasis and current population needs [1]. In addition, education and assessment have been described as needing to shift from being curriculum-driven to being competency-driven [1] to meet emerging challenges and support longitudinal professional growth. Authentic forms of education and assessment should then progressively inform mastery of competencies, rather than only indicating exam performance [2,3,4].

Competence-based education (CBE) was developed early on for medical education [5] as an outcome-based approach that aims to clarify what students should master and be assessed on. The rationale for developing competence frameworks has since been further emphasized and has been described as a potentially transformative innovation [6]. A framework may help scaffold students’ progression throughout a curriculum, guide instruction and learning activities, and provide a basis for a programmatic approach to assessment. It should also be aligned with relevant healthcare system needs and may therefore need to be adapted to the local context.

Over time, CBE has gained acceptance across many professions. In dental education, CBE has been proposed as a means to avoid ‘more of the same’ and instead ensure progression and mastery [7]. For a competence framework to be useful, it needs to be purposeful for the context in which it is used, aligned with current professional needs, and consistent with the underpinning educational approach [6,8]. Several competence frameworks exist for dental education, such as the Graduating European Dentist, from Association of Dental Education Europe (ADEE) [9], the New General Dentist framework from the American Dental Education Association (ADEA) [10], the Association of Canadian Faculties of Dentistry (ACFD) competencies [11], and the Core Competencies for Dentists in Remote Japanese Islands [12]. The latter illustrates how competence frameworks may need tailoring to local conditions. While competence-based frameworks are increasingly adopted in dental education, there is limited empirical work describing how such frameworks can be developed through participatory processes and aligned with local educational practices and assessment systems. Because frameworks must meet local needs and be recognized as relevant by teachers and other stakeholders, involvement in the development process is important [13].

As part of the development of a completely new curriculum, the need was identified for a contextually anchored competence framework that reflects contemporary developments and needs in dentistry. The intention was to use the new competence framework as a scaffold for the students to actively use to support professional development and for teachers to plan for progression across the program regarding learning activities, feedback, goal setting, and assessment.

The aim of this study was to develop a contextually grounded competence framework for undergraduate dental education through an iterative, multi-method process informed by key stakeholders.

## 2. Materials and Methods

### 2.1. Context

In 2022, a new dental curriculum was implemented at Malmö University, Sweden. The five-year dental education is underpinned by a socio-constructive paradigm where learning activities are created and interpreted within a social context to promote professional identity. It is operationalized by a challenge-based approach to learning [14], supported by collaborative learning [15] and a programmatic approach [3]. The students are introduced to the clinic during the first year and begin their clinical training during the second year. The university enrolls approximately 90 dental students yearly. Immediately after graduation, students are eligible to apply for a license and practice autonomously. Students have more clinical placements outside the university, including care for children, adults and elderly. This context shaped the need for a coherent framework that supports teachers and students’ clinical engagement and longitudinal professional competence development.

As part of curriculum development, we are seeking ways to express overarching intended outcomes in a holistic manner that would support the programmatic approach. We decided to develop a competence framework for our dental education. It is intended to be used as an overarching framework guiding progression and complementing the intended learning outcomes specified and assessed within each course. The framework should function on an overarching level, clarify the most important current and locally emerging competencies within the dental profession and scaffold the progression of learning activities and learning outcomes.

### 2.2. Study Design

**Figure 1:**
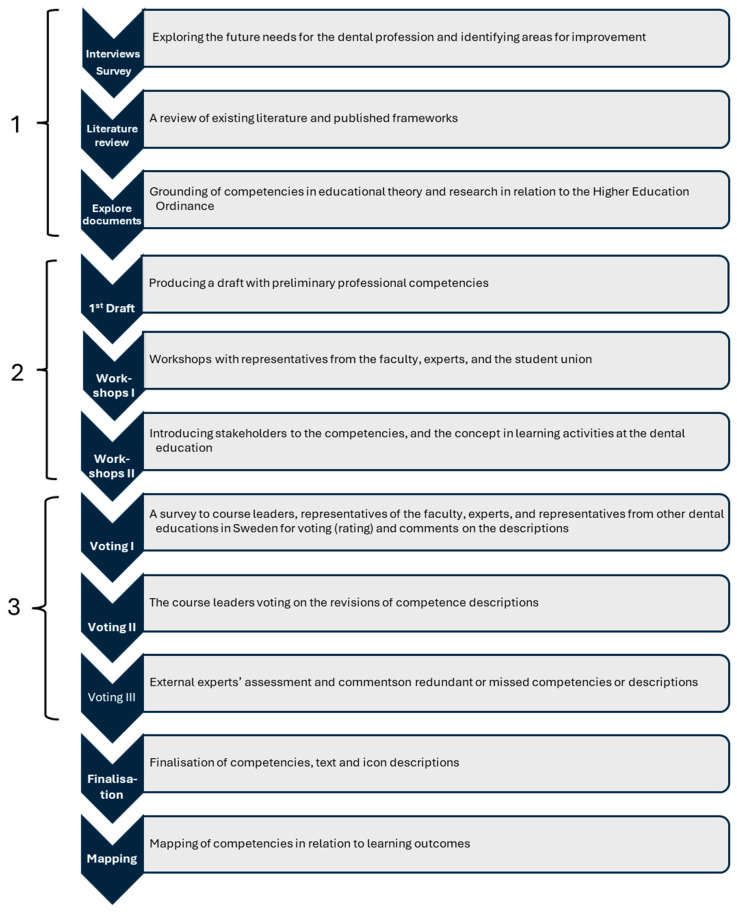
The iterative collaborative process during which the competence framework for dental education was developed. Across the three steps (1) knowledge and needs assessment; (2) drafting and exploration; and (3) relevance appraisal and final refinement, feedback informed revisions of the draft; the framework was further refined between survey rounds and then mapped to the learning outcomes.

To develop the framework, we used an iterative multi-method approach intended to support refinement and stakeholder relevance. Data were generated through document and literature reviews, stakeholder input (interviews, meetings, and workshops), and three iterative survey rounds. The phases were (1) knowledge and needs assessment (literature and document review; input from alumni, employers, and experts); (2) drafting and exploration (research team drafting followed by workshops with teachers and student representatives to identify gaps, clarify terminology, and refine descriptors); and (3) relevance appraisal and final refinement (two local survey rounds and one national external review round), with each round feeding back into further revisions of the draft before the subsequent round, including development of the visual illustration (Figure 1).

During the exploration phases, information was gathered from the literature about existing competency frameworks, from public data about sociodemographic challenges, and from surveys and conversations with experts and employers within the public sector and alumni surveys regarding opinions about needs.

The first drafts were discussed iteratively at faculty meetings with discipline specialists, teachers (dentists), and colleagues from other dental professions to explore relevance, gaps, acceptance, and feedback on the preliminary descriptions. Three rounds of electronic surveys were then conducted: two local rounds followed by a national external review round. In the surveys, each preliminary competence domain was described and rated on a five-point Likert scale (1 = not important; 2 = rarely important; 3 = neutral; 4 = somewhat important; 5 = very important). Free-text comment fields were provided for each domain description. Items with mean scores below ‘neutral’ in the first two rounds were revised. Revisions were guided by both quantitative ratings and qualitative comments and discussed within the research team.

Experts were defined as individuals with formal responsibility for teaching, assessment, or curriculum design in dental education, representing both generalist and specialist perspectives. Twenty-one teachers and seven external experts (teachers at other universities) were invited to participate in the survey. Of these, 11 teachers represented different odontology disciplines, and 10 were course leaders (eight general dental practitioners and two specialists). After identifying items rated ‘neutral’ or lower, a revision based on the free-text comments was done by the research team. During the second round, the 10 course leaders were invited to participate in a survey. As the final step, six senior teachers from the other three dental schools in Sweden were invited to respond to the survey.

### 2.3. Analysis

Descriptive statistics for the group (means and ranges) were calculated based on each respondents summarized domain ratings. Free-text comments were compiled after each round and reviewed using a pragmatic content-focused approach to identify recurring needs for clarification, missing content, or problematic terminology; these inputs informed revisions discussed and agreed upon within the research team.

### 2.4. Mapping

To explore the relation between the competence domains and course intended learning outcomes (ILOs) across the program, the research team conducted a collaborative exploratory deductive mapping analysis as an initial appraisal. The unit of analysis was the course ILOs. For each 20-week course, each ILO was reviewed and mapped to one or more competence domains when it addressed part of a domain descriptor. Interpretations were discussed within the research team until agreement was reached.

### 2.5. Ethical Considerations

According to the Swedish Ethical Review Act (SFS 2003:460), this kind of study is not subject to ethical review. The Helsinki declaration was followed; participants in the surveys were informed about the purpose and gave their consent to use the anonymous data by submitting the survey. Survey responses were anonymous, and no identifiable or personal data were collected.

### 2.6. Reflexivity

The researchers were part of a group responsible for curriculum renewal. The research group consisted of three dentists (NL, LP, SP) with long experience of clinical practice and teaching and with various roles in education at the dental school, appointed as program director, present and previous vice dean for education. The fourth researcher (CG) was a senior lecturer in medical education, working in educational development. Through ongoing discussions, we examined how our respective roles, both current and previous, and our positions shaped the interpretation of the data as well as the development process. Grounded in a socio-constructivist perspective, our varied insider viewpoints supported productive dialog and collaboration with key stakeholders (students, teachers, faculty leaders, and clinicians) throughout the development of the competence framework.

## 3. Results

Through an iterative participatory process (Figure 1), a competence framework was developed and refined.

### 3.1. Exploration and Draft

Alumni and employers reported that decision-making skills, managing a holistic view, and taking responsibility, such as for sustainability, needed to be strengthened. One member of the research team did a literature review (CG) on international competency frameworks for dental and medical education. Based on the findings in the literature (examples given in Table 1), the framework from WHO was found to be closest to the aspects brought up by stakeholders and functioned as the primary model for our first draft. The research group drafted competencies that later were elaborated on in workshops with teachers and student representatives.

### 3.2. Rounds of Revision

The process with workshops and three rounds of voting resulted in a six-domain competence framework that was progressively refined across phases based on stakeholder feedback and prioritization. After the first voting (Table 2) minor changes were required, leading to two more rounds.

### 3.3. Final Framework

**Figure 2:**
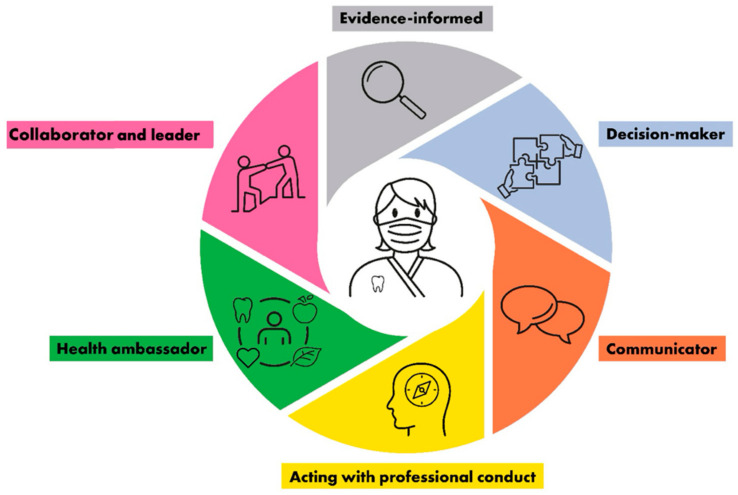
Illustration of the competence framework developed for a dental education. The framework consists of six domains, each illustrated by an icon.

The final domains were ‘Evidence-informed’, ‘Decision-maker’, ‘Communicator’, ‘Acting with professional conduct’, ‘Health ambassador’, and ‘Collaborator and leader’. Each domain was illustrated with an icon to facilitate the understanding of the concept. To illustrate how the competence domains are intertwined in the dentist profession, the illustration was in the form of a wreath (Figure 2), inspired by the WHO competence framework for universal health coverage [18].

These domains were intended to be phrased to represent overarching professional domains in the dental profession, important in our local context. Each competence domain also held a description that was agreed on, to clarify the meaning (Table 3).

### 3.4. Early Appraisal

The six domains and descriptors were agreed on and mapped to the national requirements [19]. Then, in detail, a qualitative mapping exercise was conducted to explore conceptual correspondences between the developed framework and existing intended learning outcomes (ILOs) for each course (Table 4). Areas that could be introduced early, such as ‘Evidence-informed’, were well represented during the first year. In contrast, ‘Decision-maker’ was not part of the ILOs during the first semester.

## 4. Discussion

This study contributes a systematically developed competence framework that is explicitly grounded in a local educational and healthcare context. Different competence frameworks exist and may be needed, reflecting cultural differences, national regulations, scope of practice, and variations in healthcare systems. The aim of this research was to design a purposeful, contemporary competence framework for our dental education. Such work may be valuable, as societal changes and local needs can be important to integrate into the curriculum (e.g., the shift from small single clinical practices to larger team-oriented clinics may influence the competencies required). In our study, the process (participatory, iterative, context-anchored) was considered as important as the product. The involvement of local stakeholders was intended to support acceptance, shared ownership, and application of the framework during the education. In addition, the mapping was used as an early step to explore alignment with the intended learning outcomes and potential usefulness of the framework. Agreement was reached on a final set of six competence domains with descriptors. The findings illustrate how a structured, participatory development process may support contextual relevance and educational relevance of competence frameworks, while also highlighting the need for further empirical testing in other settings.

The revision of the ‘Health Ambassador’ and ‘Decision-maker’ descriptions illustrated the importance of clarity when emerging practice expectations are made explicit. These competencies have been important previously but were expanded to better align with identified needs. Our intention was to develop a framework with clear, overarching descriptions that can be used as a scaffold for learning activities and as guidance for students across the years of study regarding program outcomes. The needs and expectations of a newly graduated dentist described by stakeholders resonated with the WHO competency framework for universal health coverage [18], published in 2022. We followed the WHO-recommended ‘adapt and adopt’ approach to develop a framework suited to our local culture and context. In our context, for example, the phrasing ‘health ambassador’ was preferred rather than ‘people-centeredness’. Some domains and expressions differ from other frameworks developed specifically for dental education [9,10,11,12]. We interpret these differences as partly reflecting contextual and cultural variation, as well as the time period when frameworks were developed.

When adopting CBE, five components have been suggested [6]: outcome competencies, sequenced progression, tailored learning experiences, competency-focused instruction, and programmatic assessment. In our context, not all components are applicable. For instance, our education is by law time-based; the progression may be partly personalized but only within the intended time frame. Our findings support the view that competence frameworks can function as organizing structures even when full competency-based education implementation is constrained.

The present framework was deliberately developed to respond to locally identified educational and societal needs, which limits direct comparability but strengthens contextual fit. There is certainly a large overlap with other competence frameworks for dental education (Table 1), but also differences. It is common to use the term ‘professionalism’ as one of the domains [9,10,11]. During our discussions, it became clear that ‘Acting with Professional Conduct’ was a preferred term in our context, for clarity and distinction between the different domains.

It appears there is less emphasis on the dimensions of ‘Collaborator/Leader’ and ‘Decision-maker’ in other frameworks for dental education [9,10]. In our context, these were competencies that employers were asking for and perceived currently being less emphasized during education.

The two domains with comments for revision in the survey were ‘Health Ambassador’ and ‘Decision-maker’. The comments may reflect professional socialization patterns in that they mirror the perception of the traditional view of our dental education, where the health-promotive perspective has been a less pronounced responsibility and mindset than desired. In our process, the voting rounds seemed to function as a bridge to increase awareness of this need. Decision-making was undoubtedly important. The concerns were about phrasing and the introduction of a new vocabulary in the description.

A competence framework should be aligned with the educational approach and the regulations on which the education is based [8]. In this study, the mapping exercises offered an initial indication of internal coherence across the curriculum. It suggested that ‘Evidence-based Practice’, ‘Collaboration’ and ‘Acting with Professional Conduct’ are introduced early, while ‘Decision-making’ is introduced later. ‘Decision-making’ may require a holistic understanding and clinical reasoning training [20], which are complex capabilities often developed during the later part of education. This mapping will be available to teachers and students and is intended to support learning progression across the study years and should be considered when changes in the curriculum are made.

Our method for developing a framework may be transferable to other contexts. The participatory process was time-consuming, but it supported acceptance and anticipated utilization. A strength was the use of mixed data sources, including research evidence, as well as perspectives from employers and teachers. Input from external experts was valuable; however, we also noted that shared local knowledge may facilitate the acceptance of terminology. Overall, our process aligns with recommended steps for competency framework development, where stakeholder involvement and consensus-building are emphasized [8]. We use the term ‘competence framework’ to describe a holistic structure, in contrast to a ‘competency framework’ that may specify knowledge, skills, and behaviors at a more detailed level.

There are limitations to this study. The work was conducted at one dental school, limiting the number of participants, and we do not claim general transferability; however, the approach may be relevant in similar contexts. Course leaders in the program are few, but were judged to be a suitable group to appraise the final nuances of the domain descriptors because of their responsibility for course design and assessment within the program.

We conducted the mapping to higher education ordinance and learning outcomes. These steps aimed to explore conceptual correspondence with the newly developed curricula and the national requirements. Since education involves many teachers that shape students’ attention, social validity and acceptance are important early steps but should be explored further. To expand on the mapping, a future step would be to map the learning activity instructions and assessments. A limitation in this study may be our positionality as researchers. We are involved in the development of education and have roles that may influence our selection of competence domains and phrasing. For this reason, it was important to use input from various stakeholders and the literature combined with reflective discussions to maintain a holistic view.

Beyond defining competence domains and descriptors, attention is needed to how frameworks are implemented, adopted, and sustained within educational systems. To make competencies explicit and observable, it is relevant to design professional activities based on a competence framework, where activities reflect the action of integrated competencies. Entrustable Professional Activities (EPAs) are descriptions of essential professional activities for general dental practitioners, and students should be entrusted to perform independently (i.e., without supervisor support needed) by the time of graduation [21]. These also need to be developed and validated in the local context [22]. Although the development of EPAs was not part of the present study, the framework provides a conceptual foundation that will inform the ongoing development of EPAs and alignment efforts. Future research should examine the implementation, usability, and educational consequences of the framework.

## 5. Conclusions

This study presents the outcome of a structured, multi-method process to develop a locally relevant competence framework for a dental education. Compared with other dental education competence frameworks, there were both overlaps and differences. The term ‘professional conduct’ was preferred over ‘professionalism’ for clarity, and ‘collaborator and leader’ and ‘decision-maker’ were described as domains emerging from stakeholder and employer perspectives relevant to this setting. Stakeholder perspectives from education and practice informed the refinement of the framework, indicating perceived relevance within this setting. Although the work was based locally at one dental school, the approach may be transferable to similar contexts.

## Figures and Tables

**Table 1:** Examples of dental education competency frameworks and the domains within each framework.

Competence Framework	Included Competence Domains
ACFD Association of Canadian Faculties of Dentistry: Educational Framework for the Development of Competency in Dental Programs [11]	Patient-centered care Professionalism Communication and collaboration Practice and information management Health promotion
ADC Australian Dental Council: Professional Competencies of the Newly Qualified Dental Practitioner [16]	Social responsibility and professionalism Communication and leadership Critical thinking Health promotion Scientific and clinical knowledge Person-centered care
ADEA American Dental Education Association: Competencies for the New General Dentist [10]	Critical thinking Professionalism Communication and interpersonal skills Health promotion Practice management and informatics Patient care
ADEE Association of Dental Education Europe: Graduating European Dentist [9]	Professionalism Safe and effective clinical practice Patient-centered care Dentistry and society Research
Japan Core Competencies for Dentists in Remote Japanese Islands [12]	Professionalism Problem solving ability based on expert knowledge Practical skills for patient care Communication skills Understanding of the role of medicine in society Scientific inquiry Comprehensive perspective on patients and consumers Ability to harness information and scientific technologies
UAE United Arab Emirates: Undergraduate Dental Competency Framework [17]	Professionalism Patient-centered care Diagnosis and management planning Clinical treatment and evaluation Health promotion Communication and social skills Scientific and clinical knowledge
WHO Global Competency and Outcomes Framework for Universal Health Coverage [18]	People-centeredness Decision-making Communication Collaboration Evidence-informed practice Personal conduct

**Table 2:** The mean and range for the rating of the domains and descriptors (Voting I) for each competence domain (17 responders). Options were rated from 1 to 5, representing ‘not of importance’, ‘rarely important’, ‘neutral’, ‘somewhat important’, to ‘very important’.

Domain	Mean (Min–Max)
Acting with professional conduct	4.7 (4–5)
Evidence-informed	4.6 (4–5)
Health ambassador	3.9 (3–5)
Decision-maker	4.4 (4–5)
Communicator	4.7 (4–5)
Collaborator and leader	4.4 (4–5)

**Table 3:** The final competence domains and descriptors. Each color corresponds to a domain shown in Figure 2.

**Evidence-informed**
Develops clinical skills to practise competently and independently.
Demonstrates knowledge of evidence-informed dental practice and research.
Applies a critical approach in professional practice.
Uses strategies for lifelong learning by seeking and evaluating knowledge, and by following and contributing to knowledge development.
Responds to changes in professional practice through a knowledge-informed approach.
Contributes to safe care and systematic quality improvement.
**Decision-maker**
Takes autonomous responsibility for decisions and actions in collaboration with the patient, while considering the team, other care providers, and society.
Makes decisions and acts with consideration for colleagues and the organization.
Applies a solution-oriented approach to problem-solving.
Adapts to unexpected or changing circumstances with thoughtfulness and sound judgement.
**Communicator**
Adapts communication to the recipient, whether orally, in writing, visually, or digitally.
Shares and conveys knowledge and marketing in an ethical and responsible manner.
Manages documentation and information sharing in accordance with applicable regulations.
Listens actively and attentively.
Builds effective relationships with patients, team members, and relevant societal stakeholders.
**Acting with professional conduct**
Acts thoughtfully, with awareness of their own limitations, and takes on new challenges with sound judgement.
Acts responsibly and considerately in ways that promote health and well-being.
Engages in lifelong learning.
Reflects on clinical practice and professional development.
Builds credibility within the profession and society.
**Health ambassador**
Designs and delivers care and health-promotion initiatives while taking the individual’s perspective into account.
Applies a holistic, health-promoting perspective to individuals and society, taking demographic, socioeconomic, and cultural factors into account.
Identifies when change is needed and acts from a resource-conscious perspective, taking health economics and sustainability into account.
Contributes actively to sustainable social and environmental development within the field of dental care.
**Collaborator and leader**
Contributes to effective collaboration and leadership by building relationships based on trust, respect, and shared decision-making.
Shares knowledge, perspectives, and responsibility, and demonstrates a willingness to learn collaboratively.
Understands their own role and respects the roles and needs of others.
Works towards shared goals and outcomes and makes effective use of collective competence.
Takes responsibility for planning and managing time effectively.
Contributes to and engages with the current organization in an informed and constructive manner.

**Table 4:** The intended learning outcomes for each semester were mapped to the competence domains to describe the relation. The numbers indicate percentage of representation of intended learning outcomes. For example, in the first semester, 76% of the learning outcomes were mapped to ‘Evidence-informed’ and 18% of the learning outcomes are related to ‘Communicator’. The darkness of the green shades illustrate variation in frequency, darker is more frequent. As each learning outcome may correspond to multiple competence domains, the summed percentage for a semester may exceed 100%. The percentages do not indicate the extent to which an outcome is taught or assessed.

Year	Semester	Evidence- Informed	Decision-Maker	Communicator	Acting with Professional Conduct	Health Ambassador	Collaborator and Leader
1	1	76		18	18		18
	2	55	10	20	30	10	15
2	3	80		10	15	10	15
	4	78	26	15	26	7	7
3	5	76	24	8	28	16	8
	6	84	32	16	37	11	11
4	7	79	21	25	17	4	8
	8	74	30	9	22	4	9
5	9	50	28	11	33	17	11
	10	42	13	21	29	29	13

## Data Availability

Data will be shared upon request.

## Abbreviations

The following abbreviations are used in this manuscript:

CBE	Competence-based education
EPA	Entrustable Professional Activities
